# Speckle Tracking Echocardiography in Pediatric and Congenital Heart Disease Patients with the Micra™ Leadless Pacemaker

**DOI:** 10.19102/icrm.2025.16103

**Published:** 2025-10-15

**Authors:** Natalia Betancourt-Guzman, Jeremy Markowitz, Erick Jimenez, Daniel Cortez, Brenda Dugas, Daniel Peck, Matthew Ambrose, Bradley C. Clark

**Affiliations:** 1 Division of Pediatric Cardiology, Masonic Children’s Hospital, University of Minnesota, Minneapolis, MN, USA; 2 Division of Cardiology, University of Minnesota, Minneapolis, MN, USA; 3 Division of Pediatric Cardiology, University of California-Davis, Davis, CA, USA

**Keywords:** Leadless pacemaker, Micra™, pediatric speckle tracking

## Abstract

Right ventricular pacing can cause electrical and mechanical dyssynchrony, potentially leading to pacemaker-induced cardiomyopathy (PICM). This pilot study evaluates speckle tracking echocardiography for the early detection of subclinical left ventricular dysfunction in pediatric and congenital heart disease patients with Micra™ leadless pacemakers (Medtronic, Minneapolis, MN, USA). We analyzed echocardiograms of eight patients (mean age, 15 years; 63% women) with Micra™ pacemakers at implant and 1 month and 1 year post-implant. Data of interest included demographics, pacemaker position, ejection fraction (EF), shortening fraction, and global longitudinal strain (GLS). The pacing percentages varied from <0.1% to 100%, with three patients having burdens of >20%. The mean GLS was −17.99, and the mean EF was 59.6%. A higher EF correlated mildly with more-negative GLS values (*r* = 0.288; *P* = .218), and greater pacing percentages correlated with poorer GLS values (*r* = −0.381; *P* = .097). In summary, GLS has the potential to identify myocardial dyssynchrony, which may have the potential to support the early detection of PICM. Further research with larger cohorts and longer follow-up is needed to validate these findings.

## Introduction

Pacemaker therapy plays a crucial role in managing bradycardia and various cardiac rhythm disorders, particularly in pediatric and congenital heart disease (CHD) patients, who often present with complex and evolving cardiac conditions. Traditionally, pediatric and CHD patients required either epicardial or transvenous pacemaker systems, each with their own restrictions and complications. Recent advancements in cardiac pacing technology, particularly the Micra™ (Medtronic, Minneapolis, MN, USA) and other leadless pacemakers, have provided additional options for patients, though their utility in patients with CHD has not been well described or defined. The Micra™ and other leadless pacemakers eliminate some of the risks related to long-term lead placements but do require larger-bore venous access and create potential for additional complications. Ventricular pacing, by bypassing the natural conduction system, results in slower myocyte-to-myocyte signal transmission and asynchronous ventricular activation.^1^ This disturbance leads to initial depolarization at the pacing site, followed by a delay in the activation of the left ventricle. Consequently, this causes mechanical and electrical dyssynchrony, manifesting as asynchronous left ventricular (LV) contractions even in patients with structurally normal hearts.

Pacemaker-induced cardiomyopathy (PICM) is defined by a decline of >10% in LV ejection fraction (LVEF) and an LVEF of <50% in patients with chronic high right ventricular pacing burdens (>40%).^2^ Global longitudinal strain (GLS) is considered a more sensitive marker for detecting myocardial dysfunction, often identifying changes before they are evident in LVEF measurements.^3^ Speckle tracking echocardiography is a sophisticated imaging technique that offers detailed insights into myocardial strain with previously published normative values in a pediatric population.^4^ This technique focuses on longitudinal myocardial fibers, which are typically the first to be affected in various cardiomyopathies. Speckle tracking may enhance early detection of myocardial dysfunction, which is particularly valuable in monitoring pediatric and CHD patients. Early identification of PICM can facilitate timely interventions and potentially reverse adverse effects.

This comprehensive analysis aimed to elucidate the impact of Micra™ pacing on cardiac function over time and identify significant associations between pacing characteristics and changes in ventricular function. We hypothesize that speckle tracking is a more reliable method for detecting myocardial dysfunction at subclinical stages, even before traditional EF measurements show a decline, in a cohort of pediatric and CHD patients.

## Methods

All pediatric and CHD patients who received Micra™ leadless pacemakers at the University of Minnesota Masonic Children’s Hospital were included in this study; no patients had the Micra™ atrioventricular (AV) leadless pacemaker. The decision to proceed with leadless pacing using the Micra™ pacemaker was made at the discretion of the pediatric electrophysiologist, and the location of the implant was chosen at the time of the procedure based on the operator’s comfort with stability and pacing parameters. Patient clinical data were evaluated, including indication for pacemaker implant, pacemaker location, ventricular pacing burden (%), and QRS duration. Echocardiograms were recorded at the time of implant and 1 month and 1 year post-implant and analyzed.

Data collection encompassed standard echocardiographic measures such as subjective ventricular function, LVEF, and shortening fraction. To measure EF on echocardiography, we used the apical four-chamber view to trace the endocardial border of the left ventricle at both end-diastole and end-systole to measure the end-diastolic volume (EDV) and end-systolic volume (ESV); when available, two-chamber EDV and ESV were used to calculate a biplane EF. The EF was calculated using the formula: EF = ([EDV − ESV]/EDV) × 100. Two-dimensional speckle tracking echocardiography was performed in the apical two-, three-, and four-chamber views. Strain measurements were performed offline using dedicated software (TOMTEC-ARENA v2.40; TomTec Imaging Systems, Unterschleissheim, Germany). GLS was calculated by the software as a composite of strain values from the three views. Pearson’s correlation coefficient was used to assess the relationship between echocardiographic measures and Micra™ parameters, and statistical analysis was performed using SPSS software (IBM Corp., Armonk, NY, USA).

## Results

**Figure 1:**
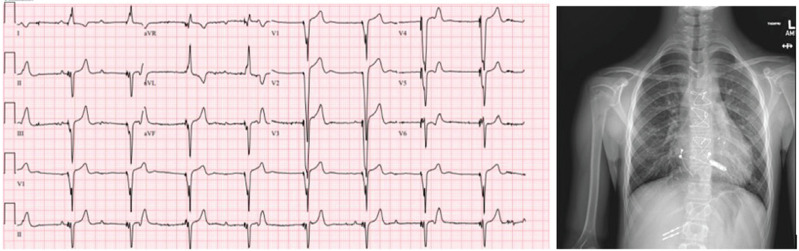
Anteroposterior chest X-ray and 12-lead electrocardiogram for a patient with repaired congenital heart disease and Micra™ implant.

We identified eight pediatric and CHD patients **(Table 1)** with Micra™ leadless pacemakers for inclusion in this study, with a mean age of 15 years (standard deviation [SD], ±4.3 years; 63% women). Among the cohort, seven had repaired CHD (88%), while one had a structurally normal heart with congenital AV block. **Figure 1** shows the 12-lead electrocardiogram and chest X-ray for a patient with repaired CHD and Micra™ implant. Six patients had the Micra™ device positioned in the septal region, and two patients had an apical placement.

**Table 1:** Patient Demographics and Clinical Information

Sex	Age (Years)	Diagnosis	Pacemaker Indication	Micra™ Site
Female	12	Pearson marrow pancreas syndrome	Complete heart block	Mid-RV septal
Male	19	Tetralogy of Fallot	Complete heart block	Mid-RV septal
Female	12	Partial atrioventricular canal defect	Sinus node dysfunction	Mid-RV septal
Male	9	cc-TGA, VSD	High-grade AV block	Mid-RV septal
Female	17	Pulmonary valve stenosis	Complete heart block	Mid-RV septal
Female	25	Tetralogy of Fallot	Complete heart block	Mid-RV septal
Male	13	Atrial septal defect, NKX2.5 mutation	Syncope	Apical–septal
Female	11	Tetralogy of Fallot and AV canal	Complete heart block	Apical

*Abbreviations:* AV, atrioventricular; ccTGA, congenitally corrected transposition of the great arteries; RV, right ventricular; VSD, ventricular septal defect.

**Figure 2:**
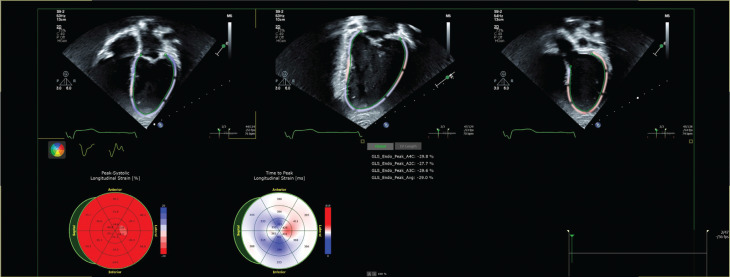
Global longitudinal strain imaging for the apical two-, three-, and four-chamber views.

The pacing percentage varied widely across the cohort, ranging from <0.1% to 100% (median, 1.42%; interquartile range, 0.23%–49.42%). Notably, three of the eight patients had a pacing burden exceeding 20%. A total of 21 echocardiograms were reviewed, with one study deemed inadequate for strain analysis. **Figure 2** and **Video 1** show images from GLS imaging. The mean LV GLS was −17.99 (SD, ±4.9), while the mean EF was 59.6% (SD, ±4.3%). **Table 2** includes the pacing parameters, pacing percentage, and echo strain values across the different time points during the study.

**Table 2:** Pacing Mode, Percentage, and Echocardiographic Findings

Patient	At Implant				1 Month Post-implant				1 Year Post-implant			
	Pacing %	Mode	LVEF	GLS	Pacing %	Mode	LVEF	GLS	Pacing %	Mode	LVEF	GLS
1	<1%	VVI 60	56	−18.1	73	VVI 60	63	−16.8	100	VVIR 70	54	−6.5
2	0.08%	VVI 40	58	−16.4	0.11	VVI 40	53	−10.8	0.23	VVI 40	56	−14
3	<0.1%	VVI 45	59	−24	0.3	VVI 45	60	−29	<0.1	VVI 45	62	−13.2
4	0.23%	VVIR 50	66	−18.3	1.35	VVI 50	57	−23	1.5	VVI 50	59	−22.5
5	41%	VVI 40	68	−16.2	33.02	VVI 40	67	−19.2	52.22	VVIR 40	66	−18.2
6	<0.1%	VVI 50	58	−18.5	N/A	N/A	N/A	N/A	N/A	N/A	N/A	N/A
7	1.24%	VDD 50-115	N/A	N/A	24.16	VVI 50	61	−19.8	27.95	VVI 50	61	−21.9
8	100%	VVIR 80	55	−14.3	99.7	VVIR 55	55		100	VVIR 55	59	−19.1

*Abbreviations:* GLS, global longitudinal strain; LVEF, left ventricular ejection fraction; N/A, not applicable.

Correlation analysis revealed that a higher EF was associated with more negative GLS values, though this correlation was not statistically significant (*r* = 0.288; *P* = .218). In contrast, a higher pacing percentage correlated with poorer GLS values, which approached statistical significance (*r* = −0.381; *P* = .097). The correlation between EF and pacing percentage was negligible, with a coefficient of −0.004 and a *P* value of .988, and there was minimal correlation between QRS duration and GLS (*r* = −0.047; *P* = .843). Specifically, among the cohort of eight patients, those with pacing burdens of >20% exhibited notably worse GLS values compared to patients with lower pacing burdens.

## Discussion

This study provides initial evidence suggesting a mild correlation between GLS and both lower LVEF and higher pacing percentages in a small cohort of pediatric and CHD patients. While its true clinical utility remains unclear, GLS may be a valuable tool for detecting subtle changes in myocardial function that are not yet reflected in traditional measures such as EF, particularly in patients with leadless pacemakers. The sensitivity of GLS to variations in myocardial strain highlights its potential role in the early detection of pacing-induced myocardial alterations, which could be crucial for timely intervention and management.

These results are consistent with existing literature that underscores the limitations of EF as an early marker of cardiac dysfunction, particularly in patients with significant pacing burdens. Traditional measures such as EF may fail to capture early functional declines, whereas speckle tracking technologies, including GLS, may detect these early changes more effectively.^5,6^ Xu et al. evaluated an adult cohort of patients and found that a lower GLS at 1 month post-implant was predictive of the development of PICM, with a sensitivity of 94%.^7^ Manocha et al. more specifically evaluated clinical risks related to PICM and found that a larger proportion of patients in an adult cohort with a GLS reduction of >15% reached a composite endpoint of heart failure hospitalization, upgrade to cardiac resynchronization therapy, or death.^8^ While these data cannot be directly extrapolated to the pediatric and CHD population, they do highlight the potential importance of strain imaging and the need to include it as a part of the long-term management of these vulnerable patients.

Our findings extend this body of work by demonstrating that GLS might be particularly useful in monitoring myocardial strain in pediatric and congenital patients with leadless pacemakers, who may experience subtle but clinically significant changes in myocardial function before traditional measures show alterations. In adult cohorts, there appears to be a lower risk of development of PICM in patients with Micra™ leadless pacemakers. Bhatia et al. evaluated over 200 Micra™ patients with follow-up data and found that nine patients (3.7%) required device replacement with either extraction or abandonment secondary to PICM.^9^ Pediatric patients and those with repaired CHD may be at a higher risk of developing PICM secondary to immature myocardium or scar related to CHD repair, especially in the setting of a high right ventricular pacing burden. Our study did demonstrate a correlation between a higher pacing burden and abnormal GLS, but long-term follow-up and additional data points will be required to evaluate whether there is a clinical correlation and a greater risk of PICM in these patients.

Our study does have several limitations. This is a pilot study with the population of leadless pacemakers chosen given their novelty in pediatric practice and the inherent lack of AV synchrony that may create an increased risk of cardiomyopathy in a vulnerable population. The small sample size, cohort of patients with lower pacing percentages, and preliminary nature of our findings mean that the correlations observed may not be fully generalizable. There was one patient included who had only one time point, but this patient was included to add to the implant strain data and was not included in follow-up measurements. Larger studies with more extensive follow-up periods and an expanded patient population including all types of pacemakers are needed to validate the role of GLS in detecting PICM and to refine monitoring strategies for pacing-related myocardial strain. As no patients developed PICM in the limited follow-up period, results cannot be truly extrapolated but can be re-evaluated over a longer period of clinical follow-up. Additionally, the cohort was limited to pediatric and CHD patients, which may not reflect the broader population of patients with leadless pacemakers. The strain measurements were performed by a single reader (J.M.) given his extensive experience with this imaging modality; future studies will include additional observers to perform inter-rater reliability as expertise improves amongst our institution. Future research should address these limitations to better understand the utility of GLS across different patient groups and pacing conditions.

## Conclusion

Our study demonstrated a mild correlation between GLS and both LVEF and pacing burden in a small cohort of pediatric and CHD patients. These observations suggest that GLS may be sensitive to changes in myocardial strain related to right ventricular pacing from the Micra™ leadless pacemaker. GLS can be a potential tool for detecting subtle changes in myocardial function that may not yet be reflected in traditional measures, such as EF in patients with leadless pacemakers. Further confirmatory studies with larger sample sizes and extended follow-up are warranted to fully assess the role of GLS in the early detection of PICM, though early results suggest the need for careful monitoring of patients with leadless pacemakers and high ventricular pacing burden.

## Supporting information

Supplementary Video 1: Live global longitudinal strain imaging for pediatric patient with Micra™ leadless pacemaker.

## References

[1] Mizner J, Jurak P, Linkova H, Smisek R, Curila K (2022). Ventricular dyssynchrony and pacing-induced cardiomyopathy in patients with pacemakers, the utility of ultra-high-frequency ECG and other dyssynchrony assessment tools. Arrhythm Electrophysiol Rev.

[2] Somma V, Ha FJ, Palmer S, Mohamed U, Agarwal S (2023). Pacing-induced cardiomyopathy: a systematic review and meta-analysis of definition, prevalence, risk factors, and management. Heart Rhythm.

[3] Pignatelli RH, Ghazi P, Reddy SC (2015). Abnormal myocardial strain indices in children receiving anthracycline chemotherapy. Pediatr Cardiol.

[4] Dallaire F, Slorach C, Bradley T (2015). Pediatric reference values and Z-score equations for left ventricular systolic strain measured by two-dimensional speckle-tracking echocardiography. J Am Soc Echocardiogr.

[5] Egbe AC, Miranda WR, Anderson JH, Pellikka PA, Connolly HM (2022). Prognostic value of left ventricular global longitudinal strain in patients with congenital heart disease. Circ Cardiovasc Imaging.

[6] Lang RM, Badano LP, Mor-Avi V (2015). Recommendations for cardiac chamber quantification by echocardiography in adults: an update from the American Society of Echocardiography and the European Association of Cardiovascular Imaging. J Am Soc Echocardiogr.

[7] Xu H, Li J, Bao Z (2019). Early change in global longitudinal strain is an independent predictor of left ventricular adverse remodelling in patients with right ventricular apical pacing. Heart Lung Circ.

[8] Manocha K, Kandola MS, Kalil R (2023). Reduction of left ventricular global longitudinal strain in patients with permanent pacemakers as a predictor of heart failure and mortality outcomes. Pacing Clin Electrophysiol.

[9] Bhatia NK, Kiani S, Merchant FM (2021). Life cycle management of Micra transcatheter pacing system: data from a high-volume center. J Cardiovasc Electrophysiol.

